# The association between plasma amyloid β and a risk of conversion from mild cognitive impairment to Alzheimer’s disease dementia

**DOI:** 10.1002/alz.091907

**Published:** 2025-01-09

**Authors:** Naoki Kaneko, Noriyuki Kimura, Teruaki Masuda, Takuya Ataka, Tsukasa Takeuchi, Temmei Ito, Hideaki Tasai, Takehiko Miyagawa, Shinichi Iwamoto, Etsuro Matsubara

**Affiliations:** ^1^ Shimadzu Corporation, Kyoto Japan; ^2^ Faculty of Medicine, Oita University, Oita Japan; ^3^ Eisai Co. Ltd., Tokyo Japan

## Abstract

**Background:**

Abnormal amyloid‐β (Aβ) deposition in the brain has been defined as the earliest pathological change of Alzheimer disease (AD). Immunoprecipitation coupled with MALDI‐TOF MS (IP‐MS) has previously revealed that APP669–711/Aβ1–42 ratio, Aβ1–40/Aβ1–42 ratio, and a combination of these two biomarkers (a composite biomarker) in human plasma correlates highly with SUVR obtained from amyloid PET. In this work, we investigate the usefulness of plasma Aβ biomarkers at baseline in predicting the conversion to AD dementia.

**Method:**

Plasma samples were obtained from participants with mild cognitive impairment (MCI) in community‐based cohort. One‐hundred seven samples measured with IP‐MS were analyzed to evaluate a risk of conversion from MCI to AD dementia. Amyloid positivity was assessed using ^11^C‐PiB PET based on a threshold of a standardized uptake value ratio of 1.2.

**Result:**

Among 107 MCI participants, 28 converted to AD dementia within seven years and 61 remained stable at the MCI state for seven years or longer. Others converted to non‐AD dementia or dropped out within seven years. The MCI participants who converted to AD dementia (MCI‐AD) showed higher baseline plasma Aβ biomarker values than stable MCI (p < 0.001). ROC analysis demonstrated high AUC (0.860, for the composite biomarker; 0.825 for the Aβ1‐40/Aβ1‐42; 0.828 for the APP669‐711/Aβ1‐42) in discriminating between MCI‐AD and stable MCI. The risk of AD conversion was significantly differed among low, intermediate and high groups categorized by the composite biomarker level (p<0.0001, Log‐rank test). In Cox regression analysis, the high and intermediate groups were associated with increased risk of AD dementia compared to the low group (the hazard ratio (HR) = 6.51 for the intermediate group and the HR = 10.33 for the high group).

**Conclusion:**

Our results suggested that the Aβ composite biomarker was associated with the risk of the conversion from MCI to AD dementia. In addition, the time from MCI to AD conversion was differed dependent on the level of the Aβ composite biomarker. The Aβ composite biomarker might be useful in predicting the conversion from MCI to AD dementia.

